# Host-directed combinatorial RNAi improves inhibition of diverse strains of influenza A virus in human respiratory epithelial cells

**DOI:** 10.1371/journal.pone.0197246

**Published:** 2018-05-18

**Authors:** Michael A. Estrin, Islam T. M. Hussein, Wendy B. Puryear, Anne C. Kuan, Stephen C. Artim, Jonathan A. Runstadler

**Affiliations:** 1 Division of Comparative Medicine, Massachusetts Institute of Technology, Cambridge, Massachusetts, United States of America; 2 Department of Biological Engineering, Massachusetts Institute of Technology, Cambridge, Massachusetts, United States of America; University of Georgia, UNITED STATES

## Abstract

Influenza A virus infections are important causes of morbidity and mortality worldwide, and currently available prevention and treatment methods are suboptimal. In recent years, genome-wide investigations have revealed numerous host factors that are required for influenza to successfully complete its life cycle. However, only a select, small number of influenza strains were evaluated using this platform, and there was considerable variation in the genes identified across different investigations. In an effort to develop a universally efficacious therapeutic strategy with limited potential for the emergence of resistance, this study was performed to investigate the effect of combinatorial RNA interference (RNAi) on inhibiting the replication of diverse influenza A virus subtypes and strains. Candidate genes were selected for targeting based on the results of multiple previous independent genome-wide studies. The effect of single and combinatorial RNAi on the replication of 12 diverse influenza A viruses, including three strains isolated from birds and one strain isolated from seals, was then evaluated in primary normal human bronchial epithelial cells. After excluding overly toxic siRNA, two siRNA combinations were identified that reduced mean viral replication by greater than 79 percent in all mammalian strains, and greater than 68 percent in all avian strains. Host-directed combinatorial RNAi effectively prevents growth of a broad range of influenza virus strains *in vitro*, and is a potential therapeutic candidate for further development and future *in vivo* studies.

## Introduction

Influenza A viruses (IAVs) are members of the *Orthomyxoviridae* family possessing negative-stranded segmented RNA genomes [1]. Wild birds are the natural reservoirs of IAVs, where they establish mainly asymptomatic infections. By virtue of their segmented genome and error prone RNA polymerase, IAVs are continuously evolving and frequent host switching is one of their prominent features. IAV makes species jumps from wild to domestic birds and various mammalian species, including humans [2] where the annual economic impact of seasonal influenza infections is substantial [3–6]. In addition to these annual epidemics, pandemic viruses emerging from host switch events have already caused tremendous losses that could be worse with the emergence of new viruses in the future [7].

Current prevention strategies for seasonal influenza involve the use of vaccines produced either in chicken eggs or tissue culture, but the time required for their production delays any large-scale prevention effort in the event of a pandemic [8]. Furthermore, the overall vaccine effectiveness for all endemic influenza strains ranged in recent years from 56 to 62% [9–11]. A universal vaccine, providing effective and lasting immunity against all subtypes and strains of IAV, is not yet available despite promising strategies and experimental data in animal models [12]. Current treatment options for influenza virus infections include two classes of antivirals, the M2-inhibitors (e.g. amantadine) and the neuraminidase inhibitors (e.g. oseltamivir). There is widespread resistance to the M2-inhibitors, and while the neuraminidase inhibitors are currently effective against most influenza strains circulating in humans [13], the potential for emergence of resistance exists and has already been demonstrated [14–18].

Due to these limitations, novel prevention and treatment strategies are needed. In recent years, a large body of literature has been published examining the interactions of the influenza virus with cellular host factors [19–24]. Several of these studies used RNA interference (RNAi) to detect host factors that influenza virus is dependent upon for successful completion of its replication cycle. These studies have produced somewhat divergent results, and most host genes identified as critical to influenza replication in one publication were not recognized as important in others [25]. Among the six, large-scale genome-wide RNAi screens, only three genes were consistently found in as many as four screens and only nine other genes were common to three screens [26]. These variations are thought to be due to different methodologies used and the occasional lack of specificity manifested by the identification of false positive hits [25, 26]. Despite these inconsistencies, these RNAi-based technologies hold promise for identifying host-directed antiviral therapeutic targets. Small molecule influenza inhibitors including verdinexor, a selective inhibitor of nuclear transport compound, have already been identified based on the results of such RNAi studies [27–31]. One important limitation of these drug development studies is the small spectrum of influenza strains and subtypes evaluated. Furthermore, there is a great potential for the emergence of novel strains, for which no vaccine has been produced and no information available regarding their susceptibility to antivirals.

The simultaneous inhibition of multiple host factors that interact with IAVs during their replication cycle may be one method of overcoming these limitations. The use of siRNA oligos as therapeutic agents has been met with challenges; however, delivery technologies are continuously improving and several candidate delivery vehicles for respiratory use have been identified [32–35]. In the experiments described here, we selected a small group of genes from the pool of hits previously identified as essential for influenza replication and evaluated them individually and in combinations in human respiratory epithelial cell lines as candidates for the prevention and mitigation of influenza infection. We evaluated the efficacy of our RNAi-based approach against a variety of human and non-human influenza strains and, due to toxicity concerns associated with suppressing host genes, we also examined the effect of RNAi treatment on host cell viability. We identified several individual and combinations of host-directed RNAi that were effective against a broad spectrum of influenza strains, meriting further investigation of combinatorial host-directed RNAi as a potential universal preventative or ameliorative therapy for influenza infection.

## Methods

### Cells and viruses

The A549 human lung carcinoma cell line (CCL-185, ATCC), Madin–Darby canine kidney cells (MDCK, CCL-34, ATCC), and the human embryonic kidney cell line 293T (CRL-3216, ATCC) were grown in DMEM media (Hyclone) supplemented with (4 mM L-glutamine, 4 mM sodium pyruvate, 100 U/ml penicillin/streptomycin) and 10% fetal bovine serum (seradigm) at 37 °C and 5% CO2. Primary normal human bronchial epithelial cells (NHBE, CC-2541, Lonza) were grown in Clonetics BEBM basal medium (CC-3171, Lonza) supplemented with BPE, hydrocortisone, hEGF, epinephrine, transferrin, insulin, retinoic acid, triiodothyronine, GA-1000 (CC-4175, Lonza). The influenza virus strains A/WSN/1933(H1N1), A/NewJersey/8/1976(H1N1), A/Netherlands/2629/2009(pH1N1), A/HongKong/8/68(H3N2), A/Nanchang/933/1995(H3N2), A/Brisbane/10/2007(H3N2), A/Wisconsin/15/2009(H3N2), A/NWS/34(HA)xA/RI/5/57(NA)(H1N2) (BEI resources, www.beiresources.org), and A/harbour seal/New Hampshire/179629/2011(H3N8), were propagated in MDCK cell monolayers grown in serum-free DMEM supplemented with 1 μg/ml TPCK trypsin (Sigma). The strains A/mallard/interior Alaska/6MP0758/2006(H10N8), A/mallard/Interior Alaska/10BM05347R0/2010(H7N3), and A/mallard/Interior Alaska/10BM02980R0/2010(H9N2) were grown in the allantoic cavities of 11-day-old embryonated chicken eggs. Viral titers were determined by plaque assay on MDCK monolayers as previously described [36].

### Selection of candidate genes for siRNA targeting

Genes identified in published genome-wide siRNA screens as indispensable for influenza virus replication [19–23] were considered for this study if they met the following criteria: genes identified as hits in three independent publications, or as inhibitors of two strains of influenza in one publication (Karlas and colleagues, 2010) and one other independent study. Using this approach, out of the 1449 genes previously identified as hits [25], only 33 genes met our criteria (Table 1). Subsequent to the design and completion of host gene selection and experiments, additional genome-wide screens [37–40] and a meta-analysis [41] have been published; however, our selection of host gene candidates was based on available data at the time of study design.

**Table 1:** Candidate genes for knockdown (those targeted in this study are in bold).

Gene	Viral life cycle target
**ATP6AP1**	vATPase complex
ATP6AP2	
ATP6V0C	
ATP6V0D1	
ATP6V1A	
ATP6V1B2	
**COPA**	COPI proteins
COPB2	
COPG	
**ARCN1**	
KPNB1	nucleo-cytoplasmic transport factors
**NUP98**	
**NXF1**	
CLK1	splicing machinery
PRPF8	
SF3B1	
SNRP70	
**RPS14**	virus-specific translation
RPS5	
RPS10	
RPS16	
B2M	miscellaneous/unknown
BARHL2	
BZRAP1	
ISG15	
JUN	
MYC	
PPP1R14D	
PSENEN	
PTPRN	
TNK2	
MAP2K3	
**PGD**	

Thirty-three candidate genes meeting the selection criteria outlined in Methods are grouped by their role and location in the viral life cycle. Genes selected for evaluation in this study are shown in bold.

### siRNA transfection

Allstars non-targeting, Allstars cell-death and unmodified siRNA oligos targeting the NP, COPA and ATP6AP1 were purchased from Qiagen. Silencer Select siRNA oligos targeting the NXF1, NUP98, ARCN1, PGD and RPS14 genes (Table 2) were purchased from Life Technologies. For short form use in this manuscript, a combination of co-transfected siRNAs is referred to as a “combo”. All siRNA transfections were performed in 96 well plates. For A549 cells, 3000 cells per well diluted in 100μl DMEM complete medium were plated. Twenty-four hours later, 0.2μl Lipofectamine RNAiMAX plus 9.8μl OptiMEM I (Life Technologies) were added to siRNA diluted in 10μl OptiMEM and incubated for 10 minutes at room temperature; complexes were then added to cells. For cells transfected with siRNAs directed at multiple genes, the final concentration of siRNA per host target and the total volume of Lipofectamine RNAiMAX per well were the same as for single gene siRNA transfections. For NHBE cells, 10,000 cells per well diluted in 100μl BEGM were plated, followed 24 hours later by the addition of 20μl transfection complexes as described above. The final concentrations of siRNA to achieve maximal knockdown for single gene and multiple gene targeting were determined in initial optimization experiments using quantitative PCR (data not shown). The final concentration of siRNA was 20nM for unmodified siRNAs and 5nM for silencer select siRNAs with the exception of siNUP98, for which 20nM and 50nM final concentrations were required for A549 and NHBE cells, respectively, to achieve optimal knockdown levels. Cells were incubated at 37 °C and 5% CO2 for 24–48 hours prior to RNA isolation, WST-1 cell viability assays, or virus infection (see below). The siRNA targeting the A/WSN/33 (WSN) nucleoprotein siNP 5′-AAGGAUCUUAUUUCUUCGGAG-3′ [42], the non-targeting Allstars siRNA, and cell-death Allstars siRNA were included in all plates as positive and negative controls.

**Table 2:** Name and characterization of siRNAs used.

Targeted Gene(s)	siRNA and sense sequence(5’-3’)	Manufacturer	Description (*http://www.ncbi.nlm.nih.gov/*)	References
Allstars	Allstars Negative Control siRNA sequence proprietary	Qiagen®	Negative control, non-targeting siRNA no homology to any mammalian gene	
NP	siNP CUCCGAAGAAAUAAGAUCCTT	Ambion®/Life Technologies™	Positive control, degrades virion RNA and mRNA	Ge (2003)
COPA	Hs_COPA_3 CTGGCGCATGAATGAATCAAA	Qiagen®	Coatomer protein complex, subunit alpha, endosomal transport	Karlas (2010) Konig (2010) Brass (2009) Shapira (2009)
ATP6AP1	Hs_ATP6AP1_7 CAGCAATGGCTCCGTCGCCTA	Qiagen®	Proton-transporting V-type ATPase, acidification of endosome	Karlas (2010) Konig (2010) Brass (2009) Hao (2008)
NXF1	s20532 *Silencer*® Select CGAACGAUUUCCCAAGUUAtt	Ambion®/Life Technologies™	nuclear RNA export factor 1 Nuclear export of viral mRNA, RNP	Karlas (2010) Brass (2009) Shapira (2009) Hao (2008)
ARCN1	s1541 *Silencer*® Select GAGAGACUCAAGAACGUGAtt	Ambion®/Life Technologies™	Coatomer protein complex, subunit delta, endosomal transport	Karlas (2010) Konig (2010) Brass (2009) Hao (2008)
PGD	s224256 *Silencer*® Select GUUUGAUGGUGAUAAGAAAtt	Ambion®/Life Technologies™	many processes including microtubule transport, cytoskeleton dependent intracellular transport	Brass(2009) Shapira(2009) Hao (2008)
RPS14	s226969 *Silencer*® Select CAAGAUUCCUCAAAAUAUUtt	Ambion®/Life Technologies™	ribosomal protein S14, initiation of translation	Karlas (2010) Hao (2008)
NUP98	s9783 *Silencer*® Select GGAUUGUUUGGAACCAGUUtt	Ambion®/Life Technologies™	Nuclear pore complex protein, nuclear import and export	Karlas (2010) Brass (2009) Hao (2008)
COMBO1	COPA/ARCN1/NUP98/ATP6AP1/RPS14	See component siRNAs		
COMBO2	NXF1/COPA/ATP6AP1/RPS14	See component siRNAs		
COMBO3	NXF1/ATP6AP1/RPS14	See component siRNAs		
COMBO4	ATP6AP1/PGD/RPS14/NUP98	See component siRNAs		
COMBO5	ATP6AP1/PGD/NUP98	See component siRNAs		
HsDeath	Allstars Hs Cell Death Control siRNA sequence proprietary	Qiagen®	siRNAs targeting human genes needed for cell survival	

### Validation of siRNA knockdowns by quantitative PCR

One day before transfection, 3,000 A549 cells or 10,000 NHBE cells per well were seeded onto 96-well plates. Transfection was performed as described above. RNA was isolated 24 hours post transfection using the MagMax 96 Total RNA isolation kit (Ambion), and cDNA was synthesized using the High Capacity cDNA Reverse Transcription Kit (Applied Biosystems). The relative amount of target mRNA was determined by singleplex quantitative PCR using pre-designed Taqman gene expression assays and Taqman Fast Advanced Master Mix (Applied Biosystems) following the manufacturer’s instructions. GAPDH was used as a reference gene, and the relative expression levels of target mRNA were normalized against cells transfected with Allstars non-targeting control siRNA. Three independent knockdown measurements were performed for each siRNA or combination of siRNAs evaluated.

### Western blots

siRNA- or mock-transfected A549 cells were washed twice with PBS, then lysed on ice using RIPA buffer containing protease inhibitor cocktail (ThermoFisher). The total protein content of cell lysates was quantified using the BCA protein assay kit (Pierce). For COPA, 10 μg of total proteins, and for RPS14, 5 μg of total proteins, were mixed with 4X Laemmli buffer (Bio-Rad) containing 2-mercaptoethanol, heated at 95°C for 3 min, then loaded onto a 10% precast polyacrylamide gels (Bio-Rad) and subjected to SDS-PAGE in Tris-glycine buffer (COPA) or run on a 4–20% Mini-PROTEAN TGX Precast Protein Gel (Bio-Rad)(RPS14). Separated proteins were transferred under wet conditions to nitrocellulose membranes (Bio-Rad), which were blocked for 1 h in SuperBlock buffer (ThermoFisher)(COPA) or using a semi-dry transfer method (Trans-Bot Turbo Transfer System, Bio-Rad) and blocked in Odyssey TBS Blocking Buffer (LICOR)(RPS14). Primary staining was done overnight at 4 °C using the following antibodies: rabbit polyclonal anti-COPA antibody (Sigma #HPA028024 at 1:500 dilution), rabbit polyclonal anti-RPS14 antibody (Abcam #ab66778 at 1:2000 dilution) and mouse monoclonal anti-GAPDH antibody (ThermoFisher # AM4300 at 1:2000 dilution). Membranes were then washed 3X in PBS-Tween (TBS-Tween for RPS14) and subjected to secondary staining using the following antibodies: IRDye ® 800CW Donkey polyclonal anti-rabbit IgG (LI-COR # 925–32213 at 1:5000 dilution) and IRDye ® 680RD donkey polyclonal anti-mouse IgG (LI-COR # 925–68072 at 1:5000 dilution). Stained membranes were then washed 3X in PBS-Tween (TBS-Tween for RPS14) and 1X in PBS (TBS for RPS14) and imaged using the Odyssey® CLx Imaging System (LI-COR). Quantitation of protein band intensity was conducted using the Image Studio Software (LI-COR).

### WST-1 cell proliferation assay to assess toxicity of host-directed RNAi

The WST-1 assay (Roche) was used to evaluate host cell viability after siRNA transfection. Twelve μl of the WST-1 reagent were added to each well containing transfected cells at specified times after siRNA transfection and incubated for 90 minutes at 37 °C and 5% CO2. Absorbance was measured at 450 nm and at the reference wavelength 690 nm using the SpectraMax M3 Multi-Mode Microplate Reader. Non-targeting siRNA Allstars and cell-death siRNA Allstars (Qiagen) were used as positive and negative controls, respectively. Cell viability was tested in three biological replicates for each siRNA or combination of siRNAs at each time point. The cutoff for viability was chosen as three standard deviations less than the mean optical density of cells transfected with non-targeting Allstars siRNA at each time point [43].

### Virus infection

Twenty-four hours after siRNA transfection, cells were washed twice with OptiMEM and infected with influenza at the specified MOIs in 50μl infection buffer (OptiMEM supplemented with 0.2% bovine serum albumin) for 60 minutes at room temperature. Cells were then washed again with OptiMEM and incubated for 48 hours (in case of the WSN virus) or 24 hours (for all other strains) at 37 °C in DMEM supplemented with 0.2% bovine serum albumin, 4 mM L-glutamine and antibiotics (A549) or BEGM with supplements (NHBE). For all strains other than WSN, 0.25μg/ml TPCK trypsin was added to the incubation medium. After the indicated incubation periods, supernatants were collected and stored at -80°C. Three independent wells were infected with each influenza strain evaluated for each siRNA or combination of siRNAs. Three independent wells for each siRNA transfected with Allstars non-targeting siRNA as the negative control, Allstars cell death siRNA, and siNP were included in every plate. To account for any variability across assays, each experimental condition was normalized against the Allstars non-targeting siRNA within the same plate. Values are reported as mean viral growth relative to the non-targeting control (a value of 1.00 denotes no treatment effect, while a value of 0.00 denotes complete inhibition).

### Data analysis

For each strain of influenza used, titers were measured by plaque assay and normalized by dividing the titer of experimental siRNA-transfected cell supernatants by the mean titer of non-targeting Allstars control siRNA-transfected supernatants for the corresponding plate. Significant differences in the relative titer from experimental siRNA-transfected and control wells were assessed using a two-tailed independent t-test. All calculations were performed using the Graph-Pad Prism software (version 5.04).

## Results

### Influenza is inhibited by knockdown of multiple cellular targets in cultured respiratory cells

**Fig 1:**
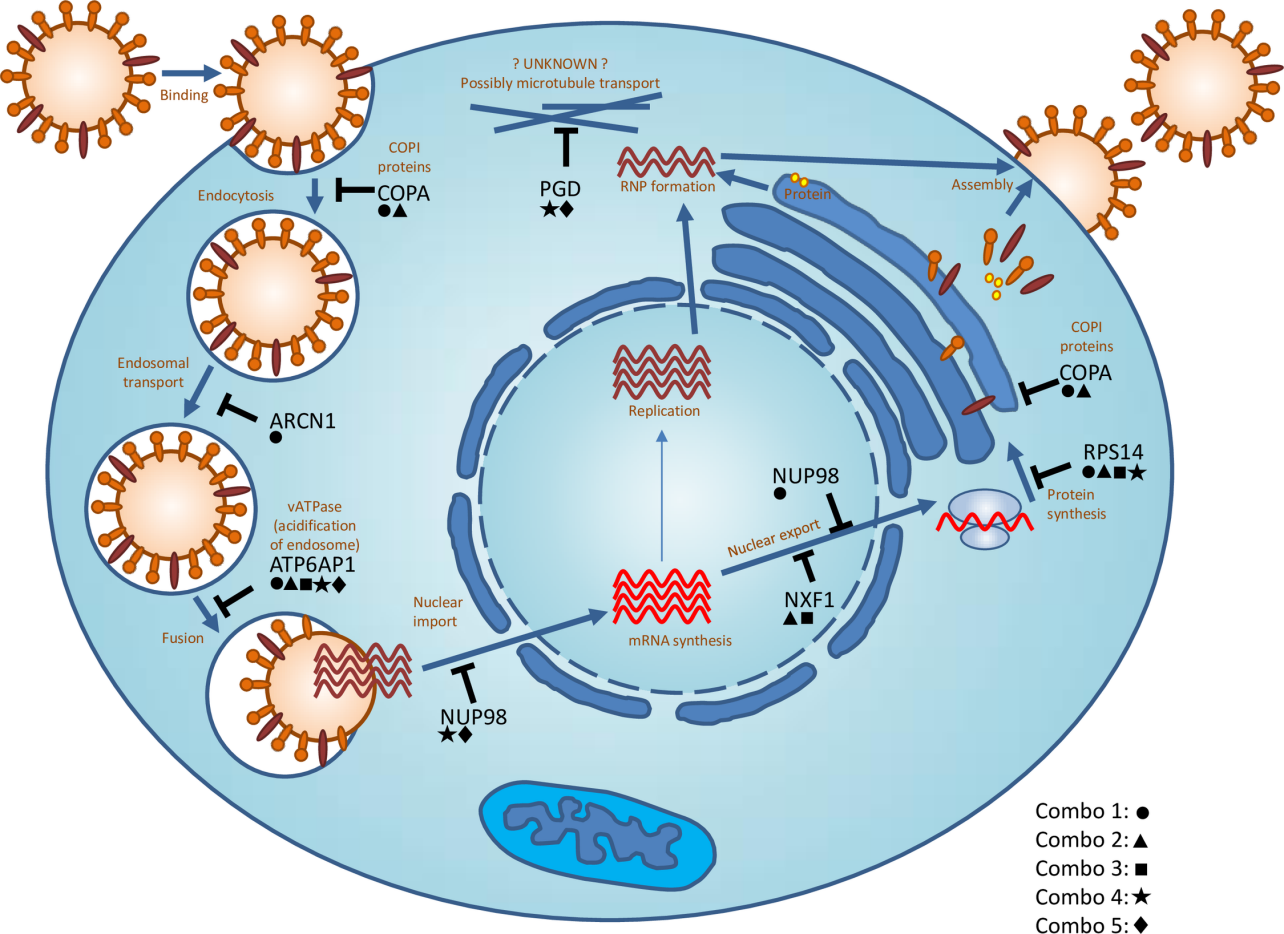
The activity of targeted host cell gene proteins in the influenza virus replication cycle. A schematic diagram showing the replication cycle of influenza virus and host genes required for influenza at different stages of the life cycle. The host genes targeted in this study are shown in black and the related part of the life cycle is shown in red. Five combinations were tested in addition to single knockdowns; the genes within each combo are denoted by symbols: combo1 (circle), combo2 (triangle), combo3 (square), combo4 (star), combo5 (diamond).

Seven representative genes among the 33 identified targets were selected as putative candidates for single and combinatorial RNAi experiments (see Table 1, bold). The selected candidates interact with influenza at multiple stages of the viral life cycle (Fig 1). siCOPA and siARCN1 impact endosomal transport, possibly interfering with endosomal transport of the internalized virion as well as protein trafficking of newly synthesized viral proteins [20, 23, 44–46]. siATP6AP1 inhibits viral fusion by impacting acidification of the endosome [47–49]. siNUP98 may impact nuclear import of viral RNA, and both siNUP98 and siNXF1 impact nuclear export of viral mRNA [25, 50–54]. siRPS14 may interfere specifically with viral translation [25]. Although the PRPF8 gene, which is involved in the splicing machinery, was reported by three independent publications as important for influenza virus replication, it was not included in our study due to the likely cytotoxic effect of its knockdown as demonstrated in prior studies [55–57]. The PGD knockdown showed no effect on viral replication in A549 cells. siPGD has been identified as required for viral replication by three independent studies and is known to impact microtubule and cytoskeletal rearrangements, though the precise mechanism of action for inhibiting influenza replication is unknown.

**Fig 2:**
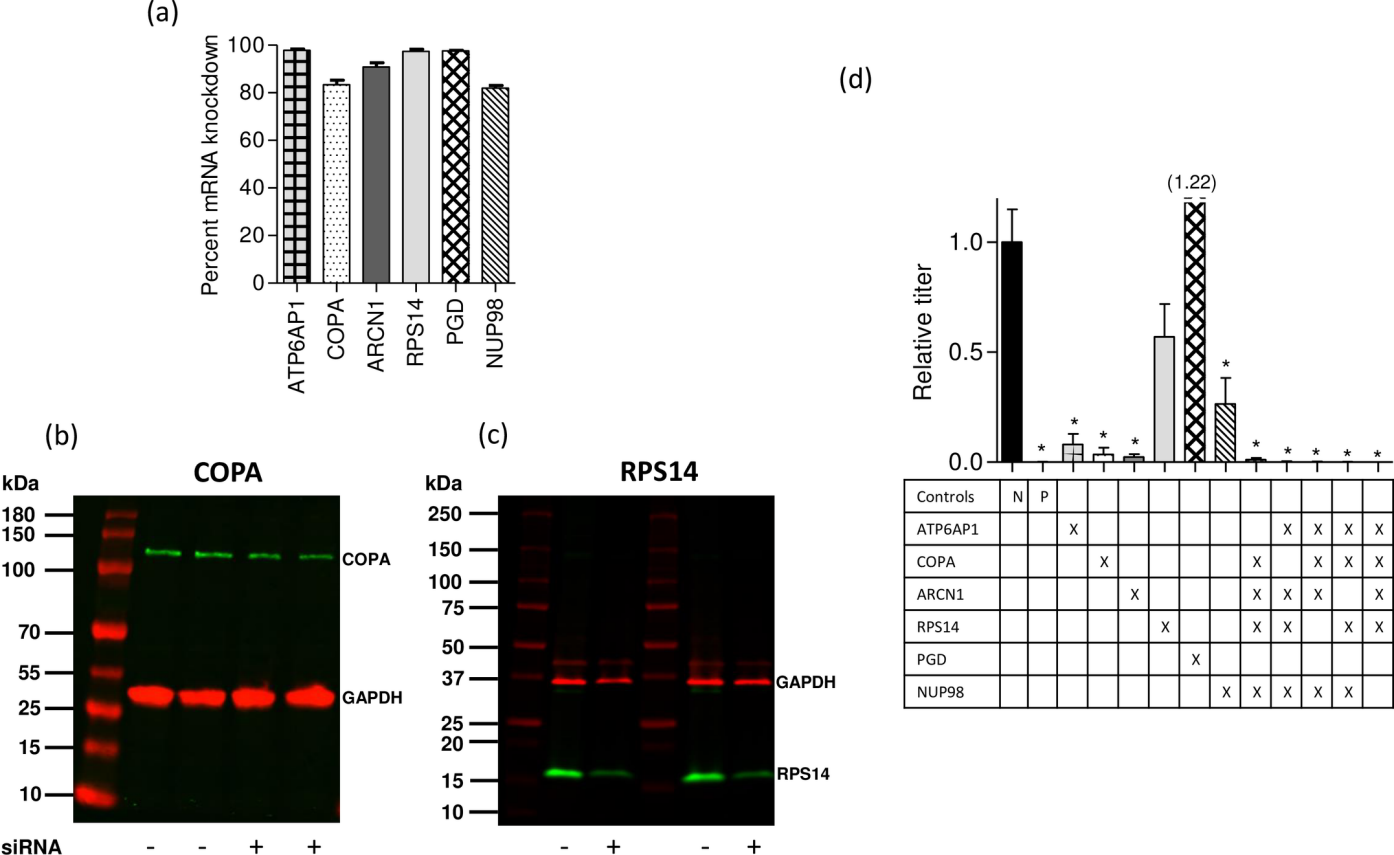
siRNA knockdown of mRNA and protein expression of targeted genes in A549 cells and the impact on influenza A/WSN/1933 (WSN) replication. (a) Knockdown efficiency of mRNA in A549 cells. Bars represent mean (+ SEM) knockdown relative to non-targeting siRNA 24 hours post transfection. (b) and (c) Knockdown efficiency of protein in A549 cells. Lysates from siRNA- and mock-transfected A549 cells were quantified using the BCA protein assay, then 10 μg (COPA, predicted size of 140 kDa) or 5 μg (RPS14, predicted size of 17 kDa) were analyzed by SDS-PAGE as 2 biological replicates. The protein band intensities of COPA and RPS14 (green) were normalized to the levels of GAPDH (red). (d) Inhibition of viral replication when genes were targeted singly, or in combination. For each bar, the targeted genes are shown (X) below. The Allstars negative control (N) and siNP positive control (P) are shown. Results are displayed as normalized to viral growth in cells transfected with non-targeting siRNA. (* p < 0.05, ** p < 0.01). A549 cells were transfected with siRNA 24 hours after plating. Cells were infected with WSN virus (MOI = 0.12) 24 hours following siRNA transfection and incubated for 48 hours prior to evaluating viral titer by plaque assay.

The selected gene targets were validated in an established *in vitro* model system for influenza infection using A549 respiratory cells and influenza virus strain WSN (H1N1). The A549 immortal human lung adenocarcinoma line has been frequently used in host-directed RNAi screens due to the relative ease of culture and maintenance of these cells compared to primary lung cells, and because human influenza viruses replicate efficiently in these cells [22, 23, 30, 31]. In this study, the mean mRNA knockdown in A549 cells was greater than 80% for six of the seven genes targeted singly (Fig 2A); attempted knockdown of NXF1 resulted in a high degree of cytotoxicity and was not evaluated further in this cell line. When multiple genes were targeted in combination, the level of knockdown attained was comparable or slightly less robust than that attained during single targeting. The weakest knockdown level observed was with ARCN1 targeted as part of a combination (67% knockdown) as opposed to singly targeted (90% knockdown). To confirm gene knockdown at the protein level, 2 representative targets (COPA and RPS14) were selected for further assessment using western blotting. After normalization to the housekeeping GAPDH protein, quantitation of band intensities revealed knockdown levels of approximately 40–60% for COPA (Fig 2B) and 55–65% for RPS14 (Fig 2C) proteins as a result of siRNA transfection.

The influenza strain A/WSN/33 (WSN) replicates rapidly in both transformed and primary human lung epithelial cells, and the genes targeted with siRNA in this study have individually been found to be essential for replication of the WSN strain in previously published experimental studies using RNAi [19, 22, 23, 30, 31]. In A549 cells, four of the six single knockdowns (ATP6AP1, COPA, ARCN1, and NUP98) resulted in significant inhibition of WSN replication (Fig 2D). The RPS14 knockdown, which disrupts viral protein translation, inhibited WSN replication but did not reach significance. The PGD knockdown showed no effect on viral replication and was not evaluated further in combinations. Viral inhibition was evaluated in five combinations of four siRNAs, targeting different parts of the viral replication pathway (shown in the table beneath Fig 2D). All combinations evaluated in this system resulted in significant inhibition of WSN replication (Fig 2D).

### Influenza inhibition in primary respiratory cells is most pronounced by knockdown of multiple cellular targets

**Fig 3:**
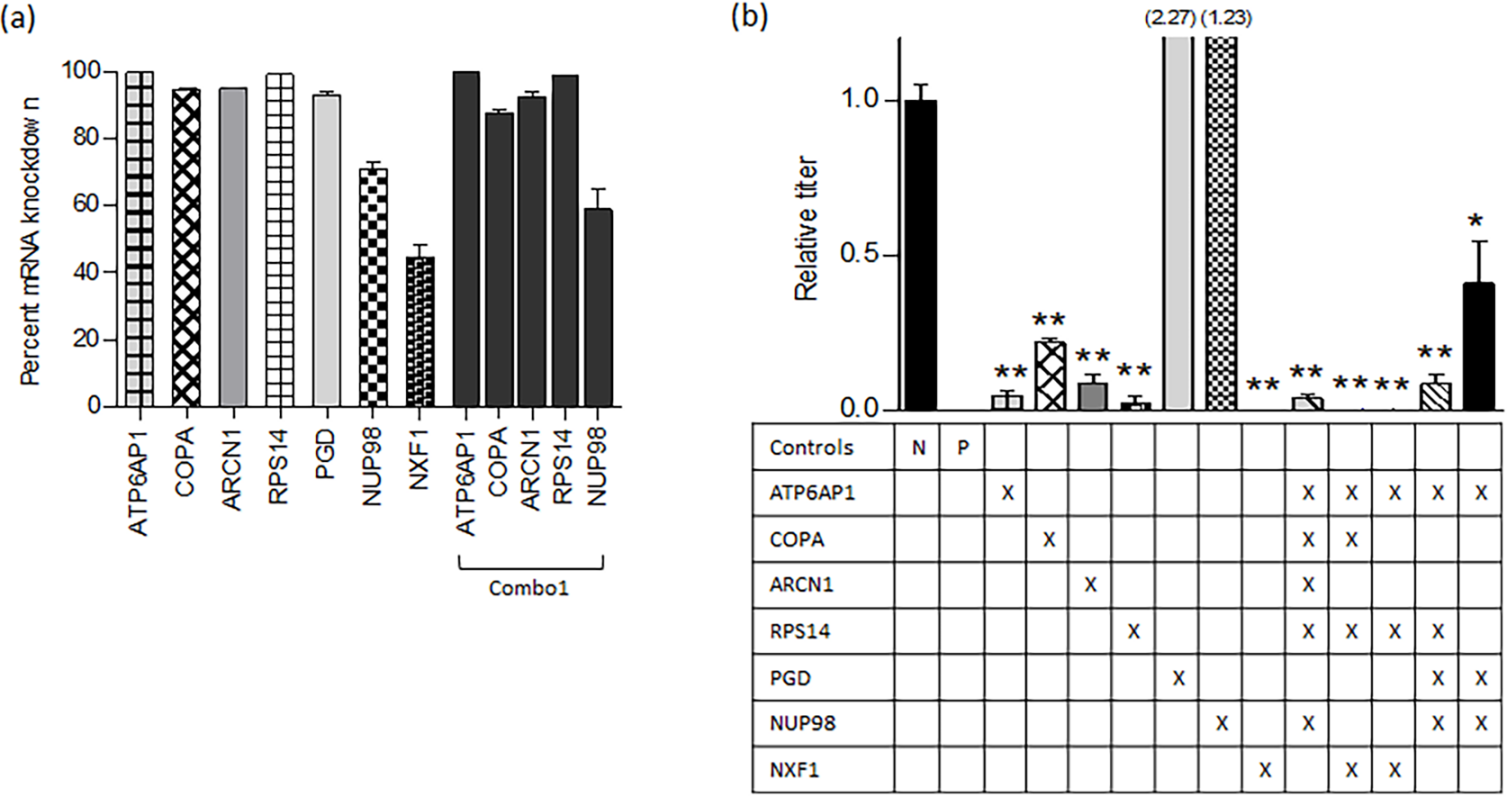
siRNA knockdown of mRNA in NHBE and the impact on influenza A/WSN/1933 (WSN) replication. (a) Knockdown efficiency of siRNA in NHBE cells; the components within Combo1 are shown as a representative example. Bars represent mean (+ SEM) knockdown relative to non-targeting siRNA 24 hours post transfection. Knockdown data for additional combinations of targets is presented in S1 Table. (b) Inhibition of viral replication when genes were targeted singly, or in combination. For each bar, the targeted genes are shown (X) below. The Allstars negative control (N) and siNP positive control (P) are shown. Results are displayed as normalized to viral growth in cells transfected with non-targeting siRNA. (* p < 0.05, ** p < 0.01). NHBE cells were transfected with siRNA 24 hours after plating. Cells were infected with WSN virus (MOI = 0.2) 24 hours following siRNA transfection and incubated for 48 hours prior to evaluating viral titer by plaque assay.

To better approximate *in vivo* conditions, including a more applicable approximation of the toxic effect of host-directed combinatorial RNAi, knockdowns were subsequently evaluated in normal human bronchial epithelial cells (NHBE) (Fig 3A). Based on the promising viral inhibition results obtained with A549 cells, we sought to explore the antiviral efficacy of additional target gene combinatorial knockdowns in NHBE cells. Most targets achieved a robust knockdown (>90%), though the nucleo-cytoplasmic targets NUP98 and NXF1 were less efficient than the other targets. The comparable knockdown levels attained for each individual component within a combination treatment are shown for one representative combination (Fig 3A, combo1).

As seen with A549 cells, most of the single targets resulted in significant reduction of WSN infection (Fig 3B). There were, however, differences between the two cell types tested; for example, RPS14 knockdown strongly inhibited viral infection in NHBE but not A549 cells, and knockdown of NUP98 was inefficient in NHBE cells despite inhibiting virus in A549 cells. As with A549 cells, the PGD knockdown showed no effect on viral replication in NHBE cells, but was evaluated in combination with other genes to determine whether the knockdown of PGD produces a synergistic effect in this context. Notably, significant evidence of WSN inhibition was once again demonstrated for all five combinations tested.

### Knockdowns that inhibit influenza are generally well tolerated in NHBE cells

**Fig 4:**
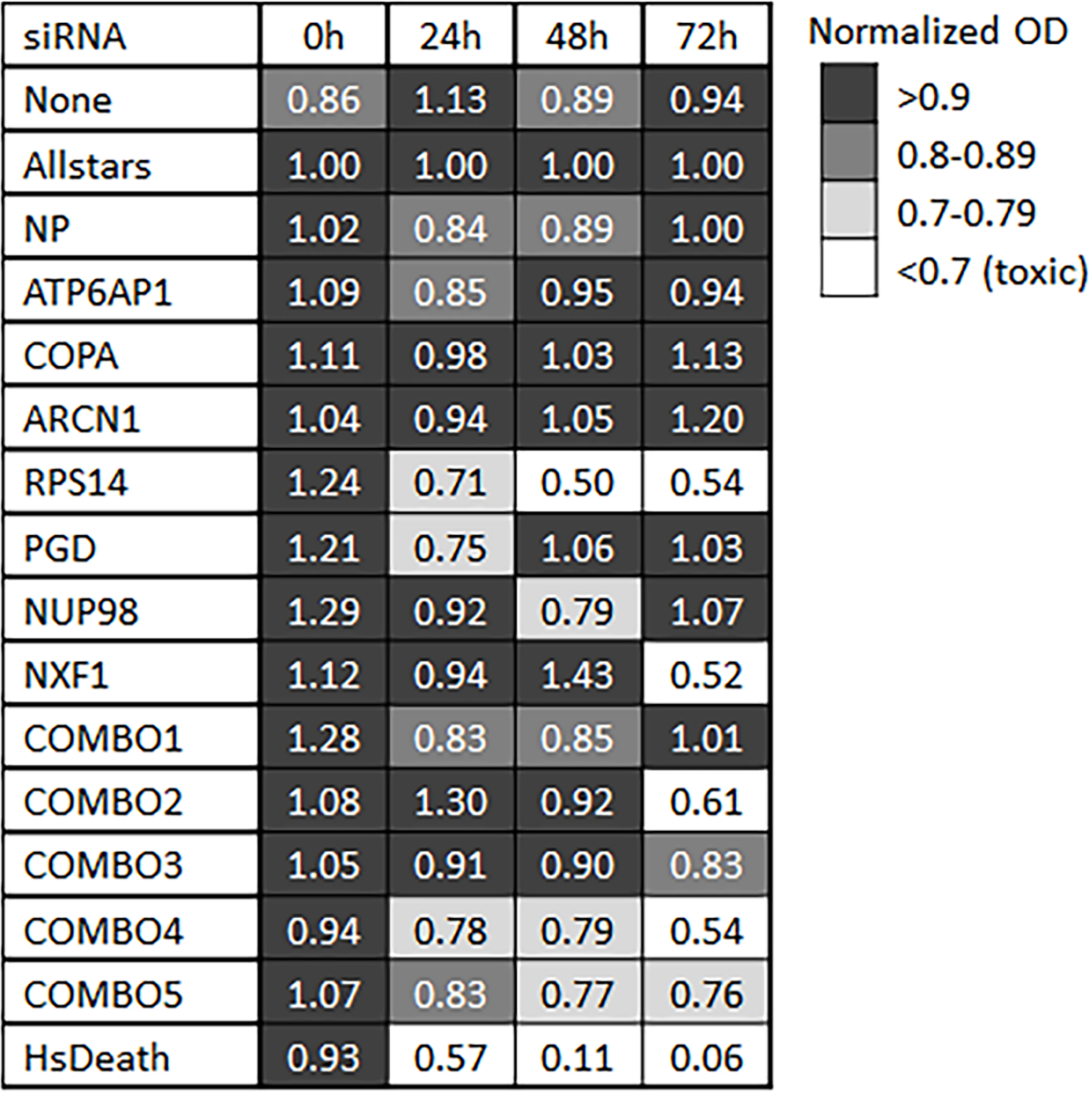
Cytotoxicity of siRNA treatment in NHBE cells. Cell viability of NHBE cells at 0, 24, 48, and 72 hours post siRNA transfection. Values are normalized to mean OD of cells transfected with non-targeting siRNA at each corresponding time interval after transfection. Relative values below 0.7 are considered toxic; all other values are considered viable, with those shaded in dark grey having the greatest cell viability (>0.9) and those in light grey being near the cutoff for cell viability (0.7 to 0.79).

NHBE cells transfected with single or combinatorial siRNA were evaluated for viability (Fig 4). After 24 hours, all cells maintained viability above the level considered toxic (70%), although RPS14 was just above the cutoff. By 48 hours, siRPS14 treated cells dropped to 50% viability, and after 72 hours, siNXF1 treated cells had poor viability, as did cells treated with combos 2 and 4. Absorbance (A450-A690) for each replicate at each time interval following transfection is presented in S2 Table.

### Combinatorial RNAi exhibit a broad-spectrum anti-influenza effect

**Fig 5:**
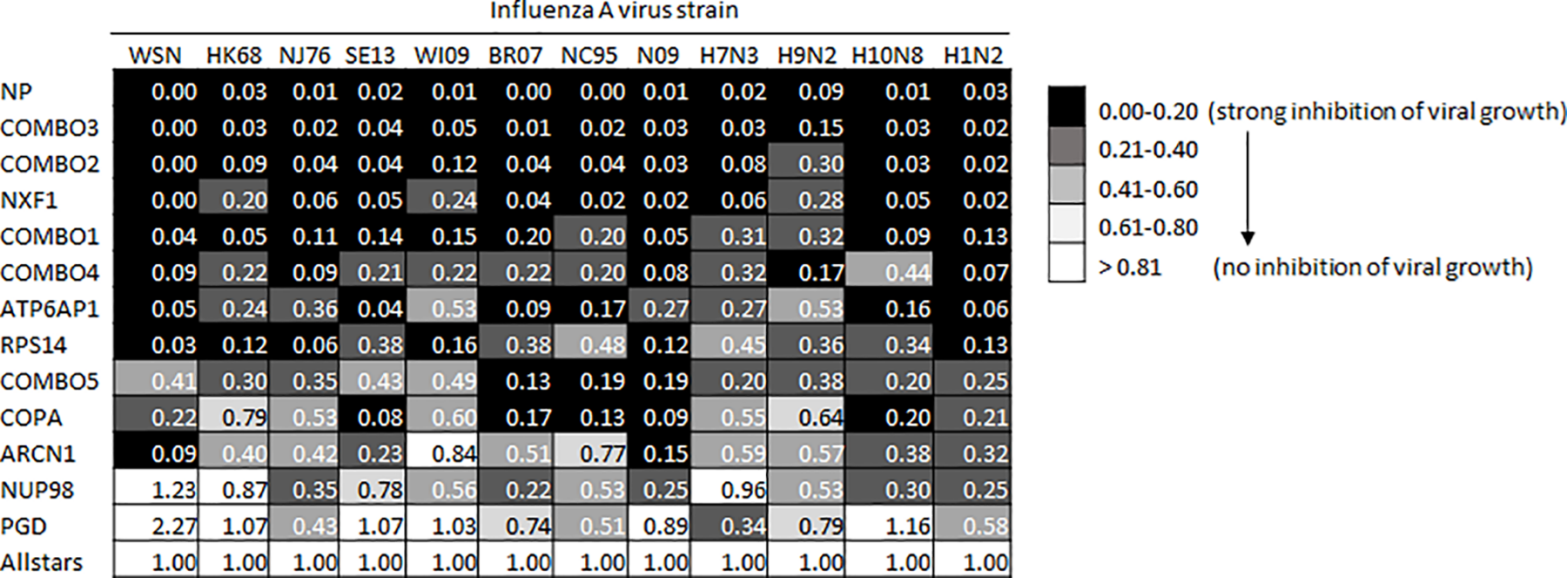
Inhibition of diverse influenza A virus in siRNA treated NHBE cells. The siRNA treatments listed on the left column were evaluated against each of twelve influenza strains noted across the top. Each combination was tested in triplicate and normalized to Allstars nontargeting siRNA. Relative mean viral growth is reported; 1.00 denotes no change relative to non-targeting siRNA, 0.50 denotes a 50% decrease in viral growth, and 0.00 denotes complete inhibition of viral growth.

To evaluate the broad-spectrum inhibitory effect of single and combinatorial RNAi on a diverse group of influenza A virus subtypes, treated NHBE cells were infected at a MOI of 1.0 with a panel of influenza viruses. It is thought that pandemic influenza could arise from the wide variety of subtypes to which human hosts are generally naïve (non-H1 or H3). However, recent events with pandemic H1N1 virus in 2009 have shown that even subtypes to which humans are exposed may emerge with passage through a variety of intermediate mammalian hosts. Therefore, while not intended to encompass all possible sources of infection or potential subtypes, we chose a small group of influenza A viruses representing human seasonal strains, a recent mammalian H3 isolate of concern, as well as a small group of diverse subtypes that have recently caused spillover infection to humans from the avian natural reservoir. In addition to the H1N1 isolate WSN, we evaluated seven additional representative human H3N2 and H1N1 strains isolated over a 50-year period, an H3N8 isolated from harbor seals in 2011, and three diverse strains (H7N3, H9N2, H10N8) isolated from mallard ducks (Table 3). Twenty-four hours post-infection, the viral titers in supernatants of infected cells were determined by plaque assay. The data is reported as mean viral growth normalized to the RNAi non-targeting control (Fig 5). Absolute viral titers for all strains and targets are presented in S3 Table. In general, the RNAi combinations demonstrated the greatest magnitude of efficacy in suppressing viral replication against the entire group of influenza strains tested. However, combo 5 was relatively inefficient, and the single target siNXF1 was a strong inhibitor on its own.

**Table 3:** Description of influenza virus strains used.

Strain designator	Strain name	Subtype
WSN	A/WSN/1933	H1N1
HK68	A/HongKong/8/68	H3N2
NJ76	A/NewJersey/8/1976	H1N1
SE13	A/Harbor Seal/Massachusetts/1/2011	H3N8
WI09	A/Wisconsin/15/2009	H3N2
BR07	A/Brisbane/10/2007	H3N2
NC95	A/Nanchang/933/1995	H3N2
N09	A/Netherlands/2629/2009	H1N1
H7N3	A/mallard/Interior Alaska/10BM05347R0/2010	H7N3
H9N2	A/mallard/Interior Alaska/10BM02980R0/2010	H9N2
H10N8	A/mallard/interior Alaska/6MP0758/2006	H10N8
H1N2	A/NWS/34(HA) x A/RI/5/57(NA)	H1N2

## Discussion

Influenza is an incredibly diverse virus that can rapidly evolve or reassort its genome content, and as frequently seen in the recent past, there is a continual potential for newly emerging zoonotic strains [58, 59]. While RNAi of a single gene (e.g. ATP6AP1, COPA, or ARCN1) inhibited replication of many strains of influenza tested here, there were several endemic strains that were not inhibited, and inhibition of diverse strains (e.g. H7N3, H9N2) was particularly weak in the absence of combinatorial RNAi. The most important advantage of combinatorial RNAi is in the potential coverage against viral escape mutants. Given the high rate of viral evolution, therapies that rely on a single step in the viral life cycle are highly vulnerable to the development of resistance; a fate well described in the early developments of antiretroviral monotherapies against HIV. These same limitations likely apply to the use of small molecule inhibitors directed against host proteins; therefore, despite promising results in experimental studies using a limited number of strains [28–31], a therapeutic strategy based on inhibition of a single host factor may only be efficacious against a narrow array of influenza strains and be prone to resistance. It is also important to note that while any therapeutic modality that is based on inhibition of multiple host factors poses the potential for toxicity in the host, toxicity is not inevitable. As seen with combinations 1 and 3 in the NHBE cell viability experiments, it is possible to combine siRNAs against multiple host genes in a manner that inhibits virus but does not adversely impact cell survival. Nonetheless, it will also be important to develop better means of assessing potential detrimental off-target effects of the siRNAs used, particularly when considering therapeutic use. It is equally important to note that while some combinations may effectively inhibit viral replication with little impact on cell viability early on, toxicity may still be an issue at later time points, a profile seen with combinations 2 and 4. Therefore, based on a relatively low cytotoxicity and high efficacy, resulting in a greater than 85% reduction in relative titer across all IAV strains evaluated, combination 3 should be considered to have the greatest potential among all of the single genes and combinations of genes used in this study for antiviral therapeutic development. However, other efficacious single gene and combinatorial RNAi should not be excluded from further evaluation based on *in vitro* toxicity data, as results may differ in an *in vivo* setting, due to the increased level of complexity represented by different cell types interacting, routes of delivery, and immune response among other.

The experiments in this study involved a limited number of host gene products. The efficiency of siRNA-targeted knockdown of these genes was assessed previously, both at the RNA [19, 20, 22, 23] and protein levels [60–65]. Based on the initial findings described here, a comprehensive assessment of potential targets and their combination is justified. The combinations included in this study ranged from three to five targets, and included only a subset of potential targets identified for each part of the viral life cycle. For example, while nuclear export appears to be an important step to target, siNXF1 suffered from cytotoxicity and suboptimal knockdown efficiency, therefore KPNB1 could be evaluated as an alternative, targeting a similar part of the life cycle. In contrast, some genes that were initially identified as potentially important targets, may not be ideally suited for inhibiting diverse influenza strains. In some instances, results of the WST-1 assay revealed cytotoxicity of individual siRNAs when used singly compared to the same siRNAs when used in combination. For example, the single use of siRNA against RPS14 and NXF1 resulted in lower cell viability relative to combo 3, which includes the same siRNA concentrations. The reason for this is not known, but may suggest a potential biological interaction between the individual gene products that warrants additional study.

Despite previous findings that the gene PGD is essential for influenza replication [19–21], knockdown of PGD did not result in any suppression of influenza WSN replication in our hands in A549 or NHBE cells. Discordant findings are common in investigations of the activities of large numbers of host genes and their effect on virus replication. Proposed reasons include differences in cell lines, viral strains, siRNA sequences, criteria to determine whether a given target is considered validated, or other difference in experimental conditions. Hao et al (2008) identified PGD as a target associated with altered influenza replication using a genetically modified influenza virus in Drosophila cells [19]; Brass et al (2009) identified PGD using A/Puerto Rico/8/34 H1N1 (PR8) in osteosarcoma cells (U2OS) [20]; and Shapira et al (2009) validated PGD using the PR8 strain in primary human bronchial epithelial cells [21]. However, it is unclear why PGD siRNA did not perform as expected in our assays.

An additional advantage of a combinatorial RNAi approach is the potential to reduce toxicity of a given target by reducing the concentration applied. Theoretically, if numerous targets in the combination are having a small effect, the importance of any one in the combination could be reduced. Alternatively, the higher concentration may be necessary to maintain breadth of activity against influenza strains. In this study, we held siRNA concentrations steady across single and combinatorial groups to avoid confounding the analysis. We therefore did not assess the impact of siRNA concentration in this study, but a more comprehensive assessment should include titration of RNAi concentrations in evaluating the potential clinical use of combinatorial RNAi.

While the current study focused on the potential use of RNAi targets against diverse influenza strains, the strategies reported here have the potential for an even broader application. The same genes that were the focus of this study on IAV have also been identified as essential host factors for the replication pathways of a number of other taxonomically distant viral pathogens. These viruses include HIV-1, Hepatitis C virus, Human Parainfluenza virus type 3, Lymphocytic choriomeningitis virus, Rotavirus, Vesicular stomatitis virus, prototype alphavirus Sindbis virus, Vaccinia virus, and West Nile virus [66–75]. The siRNA combination COPA/ARCN1/NUP98/ATP6AP1/RPS14 (COMBO 1), for example, contains siRNA for genes that are essential for the viral life cycle of each of the aforementioned viruses and studies following up our work should evaluate the effectiveness of this approach against multiple pathogens as a logical next step that would also help confirm the physiological function of these targets in the influenza life cycle. Furthermore, additional analyses, published since the conclusion of our work, have suggested additional targets that may be interacting with influenza and other viral pathogens. An example of this is URB4 [41], and it will be interesting to see how these and other host targets that we did not investigate can be used in combination to modulate host response to a variety of viral pathogens.

In addition to refining the optimal targets that maximize antiviral strength and breadth *in vitro*, host-directed RNAi faces significant challenges for development of *in vivo* animal models for study before such an approach can be pursued as a universal treatment for virus infections. These include methods of delivery as well as a more detailed assessment of off-target effects and toxicity. However, given the exquisite adaptability of influenza, and RNA viruses in general, such efforts to develop alternative therapeutic approaches are warranted. The work presented here strengthens the argument that a multifaceted treatment approach has broader efficacy than a narrowly focused one, and begins to demonstrate that it may be possible to target host gene expression to broadly inhibit diverse viral pathogens without harming the host.

## Conclusions

The results of host-directed RNAi experiments presented here demonstrated that combinatorial RNAi generally performs better than single gene targets in terms of both strength and breadth of influenza inhibition. This conclusion has important implications for influenza biology and ramifications for the design and development of siRNA as a pharmaceutical agent.

## Supporting information

S1 Table Individual and combinatorial mRNA knockdown in NHBE cells. (XLSX)

S2 Table WST-1 assay absorbance data. Measured absorbance (A450 nm-A690 nm) for all biological replicates for each target or combination of targets at baseline, 24, 48, and 72 hours following siRNA transfection.
(XLSX)

S3 Table Titers for all virus strains and targets. Absolute viral titers for all twelve influenza strains and siRNA treatments.
(XLSX)
